# Developing and Field Testing a Community Based Youth Initiative to Increase Tuberculosis Awareness in Remote Arctic Inuit Communities

**DOI:** 10.1371/journal.pone.0159241

**Published:** 2016-07-14

**Authors:** Gonzalo G. Alvarez, Deborah D. Van Dyk, Heather Colquhoun, Katherine A. Moreau, Sunita Mulpuru, Ian D. Graham

**Affiliations:** 1 Clinical Epidemiology Program, Ottawa Hospital Research Institute, Ottawa, Ontario, Canada; 2 Department of Occupational Science & Occupational Therapy, University of Toronto, Toronto, Ontario, Canada; 3 Faculty of Education, University of Ottawa, Ottawa, Ontario, Canada; Public Health Agency of Barcelona, SPAIN

## Abstract

**Background:**

Inuit in Canada have the highest reported tuberculosis (TB) incidence rate in Canada, even higher than other Canadian Indigenous groups. The aim of this study was to increase TB awareness among Inuit youth and their communities by equipping those who can best reach this population with a community based, youth focused, education initiative built on interventions adapted from a previous TB awareness study.

**Methods:**

The Taima TB Youth Education Initiative was a field test case study of a knowledge translation (KT) strategy aimed at community members who provide health education in these communities. In the first stage of this study, interventions from a larger TB awareness campaign were adapted to focus on youth living in remote Inuit communities. During the second stage of the study, investigators field tested the initiative in two isolated Inuit communities. It was then applied by local implementation teams in two other communities. Evaluation criteria included feasibility, acceptability, knowledge uptake and health behavior change.

**Results:**

Implementation of the adapted KT interventions resulted in participation of a total of 41 youth (19 females, 22 males) with an average age of 16 years (range 12–21 years) in four different communities in Nunavut. Community celebration events were attended by 271 community members where TB messaging were presented and discussed. All of the health care workers and community members surveyed reported that the adapted interventions were acceptable and a useful way of learning to some extent. Knowledge uptake measures indicated an average TB knowledge score of 64 out of 100. Local partners in all four communities indicated that they would use the Taima TB Youth Education Initiative again to raise awareness about TB among youth in their communities.

**Conclusions:**

The TB awareness interventions adapted for the Taima TB Youth Education Initiative were acceptable to the Inuit communities involved in the study. They resulted in uptake of knowledge among participants. Implementation by local implementation teams was feasible as evidenced by the participation and attendance of youth and community members in all communities. The ability to implement the interventions by local implementation teams indicates there is potential to scale up in other remote communities in the arctic setting.

## Introduction

In the 1950s, Canadian Inuit experienced some of the highest rates of active tuberculosis (TB) disease ever recorded in history [1]. Canadian public health officials applied an intensive population wide program in the eastern Canadian Arctic that involved active case finding, widespread preventative treatment, and vaccination. This intensive program along with improved living conditions is thought to have contributed substantially to the sharp decline of TB disease seen among Inuit between the 1950s and the 1980s[2]. Although the TB program achieved significant success in reducing TB among Inuit, it did so at a substantial human price[3]. Many Inuit were evacuated to southern sanatoria for long periods of time, often never to return home. Families and communities were disrupted and experienced significant trans-generational loss and suffering[4].

Present day TB epidemiology among Inuit in Canada demonstrates that the incidence rate is rising again. In comparison with other Canadians, including Canadian born Indigenous people, Inuit have a disproportionately high rate of TB. In 2012, this incidence rate in Canada was 4.8/100,000. Among First Nations people, the rate of disease was 23.7/100,000 but among Inuit it was 262/100,000[1]. Tuberculosis is a disease that figures strongly among young people. From 2002 to 2012, 31% of all cases of active disease in Canada were between the ages of 15–34 years. Over half of the active cases recorded in Nunavut from 2005 to 2010, were attributed to persons in this age group[1].

The remoteness of Inuit communities in Canada and chronic health human resources shortages present many challenges to implementing health promotion interventions. There are 30,000 people in Nunavut, and 85% of them are Inuit. Nunavut is made up of 24 small communities ranging in population size from 150 to 2,500. The territorial capital city of Iqaluit boasts a population of 7,700. None of these communities have road access and the cost of air travel to Nunavut and between communities is prohibitively expensive. In most of these communities, primary health care and health promotion is provided by community health nurses and community health representatives through the local health centre. Larger communities may have a public health nurse.

The pillars of a sound tuberculosis prevention and control program are based on prevention of TB disease and interruption of ongoing transmission of TB disease. Unique challenges exist in the prevention and control of TB in Indigenous populations. These include significant geographic dispersal across large masses of land, health care delivery challenges, the need to provide culturally appropriate care, risk factors, and the prevalence of unequal distribution of social determinants of health related to TB. In order to begin addressing any of these challenges in the prevention and control of TB among Indigenous peoples, engagement and awareness of Indigenous communities is paramount. Homegrown solutions are often the best guided and most sustainable.

In 2011, a multifaceted TB awareness, diagnosis, and treatment study [5] in Nunavut was piloted in Iqaluit, Nunavut. This project was called Taima TB-Iqaluit (Taima roughly translates as *Stop* in Inuktut). A key element of the Taima TB-Iqaluit project was a community TB awareness campaign. The interventions of the awareness campaign included the development of Inuit-specific TB education messaging, a video challenge, and community events (feasts and radio shows). Educational TB messaging was developed by local Inuit representatives and local TB health care professionals to address gaps in knowledge identified in the community (see S1 Appendix). Messages were tested by focus groups to ensure proper consideration of the historical and cultural context of TB for Canadian Inuit and to make certain that the translation to the local Inuit language was appropriate. Researchers put out a video challenge to community members to put the messaging into a relevant context in a format that supports oral Inuit tradition. The resultant videos were showcased at a community feast put on by the researchers and research partners. Radio shows were also used to communicate the messaging to the community.

In the awareness campaign, the impact on health behaviour was measured by the number of people choosing to seek testing. During the Taima TB-Iqaluit awareness campaign, the number of people attending the local public health clinic significantly increased from an average of 26 per month to 50 people per month (p <0.0002)[5].

After successful implementation of the original research interventions during the Taima TB-Iqaluit project, which included the development of Inuit-specific TB education messaging, a video challenge, and community events such as feasts and radio shows, the Taima TB team sought to expand the reach and refinement of the intervention to be used by local health care providers and educators in areas where they may have the most impact. After a review of TB epidemiology in Nunavut, and information gathered from focus groups and community members in the original Taima TB-Iqaluit project, the research team identified two primary groups who would benefit from TB awareness building interventions: Inuit youth and remote communities (outside the territorial capital). The Taima TB steering committee which is comprised of includes Inuit partners, Government of Nunavut officials, including the Territorial Medical Officer of Health, and other front line health care team members decided that the first step would be to implement the key components of the original Taima TB-Iqaluit awareness campaign in other communities in this region. The focus would be on equipping the community members who provide health education in these small communities with the tools to carry out a TB youth focused education awareness initiative. Given that sending educators to 25 communities that are not accessible by land over a giant land mass would be financially implausible not only for the present study but also for the Government of Nunavut going forward, the scalability of such an initiative would rest on whether such an initiative could be done by local implementation teams.

The Taima TB Youth Education Initiative was designed to determine whether the interventions developed for the original Taima TB-Iqaluit awareness campaign could be adapted to Inuit youth living in isolated communities, and to ascertain the feasibility of implementing these interventions in remote settings with limited resources. The first stage of this knowledge translation (KT) study was to determine the barriers to TB awareness among Inuit youth in isolated communities and adapt the original KT interventions to address these barriers. The second stage was a test of implementation comprised of a field test by the research team followed by an application test by local implementation teams. The effect of the adapted interventions was measured in terms of acceptability, knowledge uptake, and health behavior change. The feasibility of implementation was determined by the ability of implementation teams to effectively deliver the interventions.

## Methods

### Design of study

A field test case study using a knowledge-to-action process [6] building on the original research interventions from the original Taima TB-Iqaluit project. There were two stages to the Taima TB Youth Education Initiative:

1. Adaptation: Adapting the interventions from the original Taima TB-Iqaluit study
2. Implementation:
  - Field test of the adapted interventions by the research team
  - Application of the adapted interventions by local implementation teams

#### Adaptation

The Taima TB Youth Education Initiative interventions were adapted to be particularly acceptable to Inuit communities in addressing the unique linguistic, social, and historic context of tuberculosis in the Canadian Arctic. They were also adapted to overcome barriers that exist in reaching Inuit youth with health promotion learning.

The original Taima TB-Iqaluit project[5] produced culturally appropriate materials including the five messages (see S1 Appendix) that were tested during the awareness campaign. To ensure the materials were sensitive to Inuit culture and tradition, Inuit health care workers as well as a focus group of Inuit lay people from the community were included in developing the key messaging/topics to be discussed. The messaging was tested with lay people for appropriateness and on learning that much of the Inuktitut language used was far too formal it was modified to be more colloquial. The messaging also reflected the historical issues surrounding TB in the Canadian arctic where many people in the 1950-60s were sent south to TB hospitals for years at a time and in some instances died in these far off places without family support. This history is reflected in the first TB message: “TB is treated here in Nunavut and is curable”. The messaging in the original Taima TB-Iqaluit project resulted in a significant number of people coming to get tested for TB. The people who came to get tested did so of their own accord suggesting that the messaging was suitable for reaching the community. This messaging was used as the foundation of the Taima TB Youth Education Initiative.

To address the important historic context of TB in this population, the youth learning interventions include inviting elders from the community who have had TB in the past to share their experiences with the youth in Inuktitut. Inuit history is one of an oral tradition where stories are passed orally from one generation to another. Teaching is passed on orally in community rather than by the written word. The focus group from the original Taima TB-Iqaluit project also identified a preference of Inuit to learn health information from other Inuit. The video challenge part of the intervention was designed to allow participants to consolidate their learning by sharing with their community in an oral/visual medium and providing a venue for Inuit to learn from Inuit. The videos would be screened during a community feast dedicated to the study. The community feast is quite a traditional event where people come to share traditional foods, knowledge, and experiences.

Barriers to reaching youth in these communities were identified through Taima TB-Iqaluit focus group, feedback from local health care providers, feedback from the Taima TB-Iqaluit research team, and consultation with a youth development specialist. Adaptations to the original Taima TB-Iqaluit TB awareness interventions were made by the research team to address these barriers. The interventions were further refined following the field test to overcome identified barriers and limitations while accentuating strengths of the interventions for application by local implementation teams.

The core of the Taima TB Youth Education Initiative was made up of three activities: (1) Youth Learning Activities, (2) Video Challenge and (3) Community Events. The adapted interventions were packaged in a way that could be implemented by local health care providers and educators. This included a ‘how to’ manual outlining how to plan and implement the youth activities, video challenge, and community event, and sample videos to aid with the video challenge[7]. Flexibility was built into the Taima TB Youth Education Initiative KT intervention project in order to respond to the unique strengths and needs of each community while maintaining a core set of research activities that was consistent and reproducible.

#### Implementation

Two implementation strategies of the KT intervention were applied in this project in a sequential fashion. The plan was to implement the initiative in 5 communities. A research team would visit two communities to field test the adapted interventions. Following the field test, the initiative would be applied in three additional communities by local implementation teams.

The youth learning activities were developed to provide youth with the opportunity to learn about the disease and consolidate their learning by making a video to teach their community about it. The concept of 5 hands-on learning activities to correspond to the messaging developed for the original Taima TB-Iqaluit project was developed in consultation with a youth development expert on the optimal youth learning strategies. Building on the principle that youth learn best from hands-on, experience based activities[8], the implementation teams facilitated their engagement through active learning stations related to the ‘5 TB Facts’. The learning stations were set up to allow them to interact with the messaging in their own context (eg, hunting/fishing, multimedia, experience of Elders in their community, games, skits). Minor adaptation of the activities was ongoing as the products were continually refined for ease of use, fitting into timeframe, appropriateness to audience, and availability of materials in a small remote community. The video challenge followed the youth learning activities

The community event was hosted by local partners in each community to give community members an opportunity to celebrate the results of the work done by the youth. These events include TB teaching through the youth videos and community/public health nurses, and the sharing of TB experiences by elders. As per Inuit traditions, local country foods were served at these events. Given the popularity of radio among older community members in the Arctic, call-in radio shows about tuberculosis were scheduled in each community as a lead up to the community events. The Taima TB Youth Education Initiative includes these community events as an important vehicle for knowledge sharing.

### Settings and Participants

#### Communities

The communities invited to participate ranged from 500–1900 in size and had moderate to heavy burdens of TB disease. Communities were invited based on the following criteria:

1. Number of active TB disease cases (absolute and rate per 100,000) over the past 10 years
2. Current or emerging TB issues
3. Lack of existing TB resources in the community
4. Willingness of the community to participate

The Taima TB steering committee comprised of Inuit representatives, Government of Nunavut representatives, including the Medical Officer of Health, and senior public health nurses, were presented with a list of communities that met the first criteria. The committee prioritized these communities based on criteria two and three. Regional and community health care professionals, administrative officers, community leaders, and health committees in these communities were approached to determine which communities fit the final criteria.

**Fig 1:**
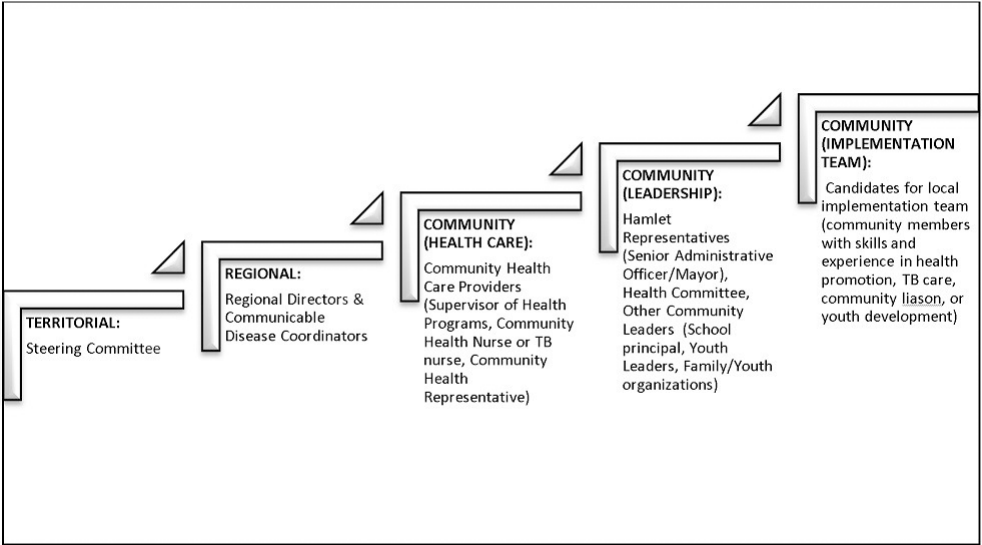
Community Engagement. Process by which community leadership was engaged for this study.

Fig 1 outlines the process by which leadership was engaged. Input and approval was sought from Inuit and government partners at the territorial and regional level prior to approaching community leadership. Communities were invited to participate via their administrative and health care leadership. They identified local implementation teams. The local implementation teams were actively involved in the adaptation, implementation, and evaluation of the intervention.

#### Implementation teams

There were two types of implementation teams: the research team and the local implementation teams. The research team included the Taima TB program director/TB physician, the project coordinator/public health nurse, a youth facilitation specialist, and Inuit organization representative, all from outside the communities. The research team was supported in each community by local partners and relied heavily on their context expertise.

Putting together a local implementation team with the proper blend of skills and experience was integral to the successful implementation in each of the communities. Based on consultation with regional and community health care providers, the research team identified people in remote communities who would be best positioned to implement the health promotion interventions. In order to capitalize on the unique mix of human resources available in each community, the research team placed emphasis on recruiting local implementation teams based on a set of skills and experience rather than job description. The research team identified three key skill sets for successful implementation:

➢ community liaison experience (community health representative, health committee member, or TB assistant)
➢ TB content knowledge (TB nurse, or Community Health Nurse), and
➢ youth facilitation skills (teacher, youth worker, or community health development coordinator).

#### Participants

Youth between the ages of 12 and 20 were invited to participate. The recruitment process and type of venue for the activities varied in each community as per community preference. The project sought to capitalize on the different types of youth development resources that were available in each community. No remuneration for participation was offered to participants.

The Ottawa Health Science Network Research Ethics Board (OHSN-REB), formerly known as the Ottawa Hospital Research Ethics Board, approved the following consent process as part of the ethics approval of this project (#20120797). In accordance to their guidelines for obtaining consent for research with minors [9], the OHSN-REB agreed that parental consent was not required for participation in this study. The OHSN-REB guidelines state that if an adolescent “can understand the information & appreciate the relevance of the decision being made” the adolescent can provide consent for their own participation and no parental consent is required. It was reasonable to expect that youth participants in this project would be able to provide consent for themselves, particularly given that this initiative is purely educational and is not invasive in any way. Participants were asked to review the information sheet and sign a OHSN-REB approved consent form at the first session. Parents or other community members with questions or concerns were given the opportunity to contact the community liaison or another member of the Taima TB team. All consent documents were available in English and Inuktut.

### Analysis

Evaluation of the adapted interventions with Inuit youth in remote communities focused on the acceptability of the interventions, knowledge uptake by participants, and health behaviour change in the community. Feasibility of implementation of the interventions by local implementation teams was also measured.

### Outcomes

Acceptability: Local community members and local health care workers completed a questionnaire to evaluate the educational videos for ease of use, and acceptability. The evaluation questionnaire was available in Inuktitut and English. The questionnaire was administered at health committee meetings or community events after viewing footage produced during the youth activities.

Knowledge Uptake: Knowledge uptake was measured through participant interviews that occurred following the last education session. A video interview of a random subsection of project participants were asked a pre-specified set of questions about what the content of the key messages that they learned during the learning activities. Each video was scored by two of the investigators separately and differences in scores were adjudicated by consensus.

Health Behaviour Change: The indicator of health behavior change was the difference in the number of people presenting to the health centre for TB testing pre- and post- intervention.

Feasibility: The number of youth recruited and their continued presence over the three scheduled sessions was the first measure of feasibility of engaging youth to this type of learning activity. The second measure of feasibility was the number of people who came to the community feast from the community at large. The third measure of feasibility was the ability of research team and local implementation teams to implement the core activities as planned. A final measure of feasibility was to determine if similar results could be achieved by implementation teams made up of community members with only remote support from the research team.

## Results

### Adaptation

The identified barriers to implementing the research interventions with Inuit youth in remote communities fell into two categories: barriers to youth learning and barriers to local implementation (Table 1). Major adaptations to the original interventions to overcome these barriers included the development of the culturally sensitive, age appropriate learning stations and the structuring of local implementation teams (Table 1).

**Table 1:** Barriers to application of original research interventions.

Barriers	Corresponding Adaptation of Research Interventions
**Barriers to Learning**	
Teaching model used (didactic traditional classroom style)	Consultation with a youth development expert to develop activities based on experiential, interactive, hands-on learning model
Lack of Inuit-specific learning materials	Elder participation in the learning stations; Use of Inuktut as much as possible; Expand use of oral/visual media to support storytelling tradition and the oral tradition of knowledge sharing; Facilitate a community event for information sharing
Variation in learning styles	Youth can apply what they learned from the youth activities while making the videos
Reproducibility of interventions	Development of core interventions (learning activities, video challenge, community events) based on consistent messaging while maintaining flexibility in implementation to allow for the uniqueness of each community
Health care professionals doing the teaching	Videos created by youth who are familiar to community members may attract attention to learning
Limited reach of pamphlets or posters	Use of a video of a community member (youth) discussing key TB facts when teaching another community member
Negative stigma related to TB	Celebration of products produced by youth combined with traditional Inuit feast
**Barriers to Implementation**	
Remote locations	Repackage interventions for implementation by a local team
Unsustainability	Repackage interventions for implementation by a local team; Ongoing use of videos as a learning tool
Limited physical resources	Activities designed to require few resources which can be obtained easily in northern communities
Limited human resources	Build local implementation team based on interest in teaching/health promotion and a set of skills needed for implementation rather than job description
‘One size fits all’ approach	Development of a core set of interventions (learning activities, video challenge, community events) based on consistent messaging while maintaining flexibility in implementation to allow for the uniqueness of each community

### Implementation

The four communities that participated in the Taima TB youth initiative varied in population size, TB burden, governance, and infrastructure for health promotion, youth development, and education (Table 2).

**Table 2:** Participating Communities.

Community	Population	Inuit	Youth (10–19)
**Community #1**	900	95%	23%
**Community #2**	1400	95%	22%
**Community #3**	1900	92%	22%
**Community #4**	500	95%	19%

#### Feasibility

Community Participation: One measure of feasibility was the ability to implement activities as planned. Six communities were invited to participate in two regions of Nunavut. One community declined, citing a lower burden of disease and other health promotion priorities. Five communities recognized a need for TB education within their communities and agreed to participate. One of these communities struggled with initiating the community engagement process due to high staff turnover and an unusually heavy burden of disease. They were unable to implement the initiative prior to the project end date. The other four communities were able to complete the community engagement process from the invitation to participate to implementation and evaluation.

Youth Engagement: The participant recruitment strategy, venue for implementation, and type of facilitator varied in each community based on available human and logistical resources (see Table A in S2 Appendix).

Table 3 describes the youth participants. In each community, the youth events and video challenge were held over 3–4 sessions of approximately 90 minutes each. Attendance by youth remained consistent or increased over the course of the education sessions (see Figure A in S2 Appendix). The participants remained engaged over the course of the three planned activities as evidenced by the fact that overall the participation did not decrease over the 3–4 sessions in all four communities.

**Table 3:** Youth participants.

	Implementation Team	Total # of Participants	Gender	Ave. Age	Age Range
**Community 1**	research	14	M = 6 F = 8	15.4	13–21
**Community 2**	research	9	M = 6 F = 3	17.8	13–21
**Community 3**	local	12	M = 7 F = 5	17.5	17–18
**Community 4**	local	6	M = 3 F = 3	12.5	12–13
**TOTAL**		**41**	**M = 22 F = 19**	**15.8**	**12–21**

Three out of four of the communities (1 research team and 2 local implementation teams) produced useable footage clips but cited time as the primary constraint to finalizing complete, stand-alone videos. The group in the community that was unable to produce useable footage was challenged by a wide variation in age and skill levels within the group. Feedback from facilitators indicated that the younger groups from the smaller communities needed much more support from facilitators to produce coherent footage.

Community Events: While each local implementation team delivered the same messaging at their community event, each event was different based on the practices in each community and strengths of the local implementation teams (see Table A in S3 Appendix). A total of 271 community members attended the community gathering across the 4 communities. Community participation ranged from 4–8% of the total population of these communities (see Figure A in S3 Appendix). Call-in radio shows were done in each community. Approximately 5–10 questions were called into each hour long show. The radio shows were in both English and Inuktut.

#### Acceptability

Six health care workers and 11 community lay people answered the questionnaires. All of health care workers and community lay people surveyed reported the Taima TB youth initiative acceptable for this community to some extent (Table 4).

**Table 4:** Questionnaires Answered by Community Members and Local Health Care Providers (n = 17).

	Not at all	To a small extent	To a moderate extent	To a large extent
*Do you think that a video is a useful way of learning about a health concern like TB*?			7	9
*Do you feel that the videos that you watched are relevant to life and culture in your community*?		1	6	9
*Did you learn something from the videos that you watched*?		2	5	9
*Would you recommend that these videos be used for future teaching about TB in your community and other communities in the region*?		1	4	11
For Health Care Providers: Do you feel that the videos add to or enhance uptake of TB knowledge among participants?		1	1	4

#### Knowledge uptake

Knowledge uptake in the second community visited by the research team was 78%. In the communities were implementation was done by local implementation teams was 54% (53% in community 3 and 55% in community 4). Evaluation challenges in the first community to participate in the initiative meant that results are not available for this community. Diverse literacy skills within this group made reliable written and video footage evaluation difficult. In order to address this challenge, after the first community, the evaluation process was adapted to include the video interviews used in the other three communities. For a breakdown of knowledge uptake by interview question see Table B in S2 Appendix.

#### Health behaviour change

Similar to the original Taima TB-Iqaluit project, the intent was to measure the impact of the interventions on health behaviour in the community by looking at the number of people presenting to the health centre pre and post intervention. Data for people presenting to the health centre for TB testing (passive screening) proved to be impossible to obtain in three of the four communities. Many of the health centres in the project communities do not collect this data or do not distinguish in their data collection between passive and active testing.

## Discussion

In four communities in Nunavut with populations ranging from 500 to 1900, over 40 youth participated in the Taima TB Youth Education Initiative. The initiative was first implemented by the research team and then independently by local implementation teams with support from the research team remotely. Knowledge uptake by the youth participants was moderate across the communities. In communities where the initiative was implemented by local health educators, knowledge uptake scores were lower, but still satisfactory. Scores were better in the older youth groups compared to the younger youth. The acceptability of the interventions was underscored by the questionnaire completed by lay community members and local health care workers. This suggests that the videos were a useful way of learning about TB, were relevant to this milieu, offered new information, and could be used for future TB teaching. These interventions are feasible as evidenced by the participation of youth and community members as well as the sustained presence of youth in each community over the 3 sessions in each initiative. Similar youth participation levels were seen during the field test by researchers and the implementation by local partners. Activities were implemented as planned in all participating communities.

The original Taima TB-Iqaluit study focus group identified Inuit youth as an important target for interventions that enhance TB awareness. In their study of knowledge translation strategies in an Inuit community, Pufall et al. [10] also highlighted the importance of involving youth in delivering health messaging. Knowledge uptake and sustained youth engagement throughout the initiative indicates that the Taima TB Youth Education Initiative demonstrates a concrete example of how both enhancing health knowledge among Inuit youth and involving them in delivering health messaging may be successfully achieved. The creative process of video making creates a positive learning environment and builds confidence in youth in their ability to contribute to the improvement of health in their communities [11]. McShane et al.’s [12] study of health information dissemination strategies in an urban Inuit community points to the need for knowledge products that incorporate Inuit oral traditions for knowledge sharing. These findings are supported by the community members and health care providers surveyed as part of the Taima TB Youth Education Initiative study, who indicated they found the videos produced as part of the interventions were a useful way to receive health information in a way that is acceptable for their communities.

Overall the KT interventions proved to be feasible as implemented both by the research team and independently local health care educators. Only one out of five communities was unable to complete the initiative because of a high staff turnover and an unusually heavy burden of disease at that time. Two communities were able to successfully complete the implementation of the initiative on their own. They did all the activities, had good attendance, and produced similar products to the communities lead by the research team. Achievability of the video challenge may depend on the age of participants and possibly on the size of the community. The two larger communities in the project (one in the research team group and one from the local implementation team group) also had older participants. The groups with older participants produced useable, stand-alone TB learning video footage, while the younger groups needed more support from facilitators to produce good footage. Feasibility of the implementation of community events was evidenced by the high attendance rate in each community. Each local implementation team indicated that they would recommend the use of the Taima TB Youth Education Initiative approach going forward.

### Lessons Learned

This research could provide significant benefit to other researchers looking to learn from the experience of the Taima TB research team. In regards to lessons learned, Table 1 shows the identification of barriers to learning and to implementation with the corresponding adaptations is a valuable resource to improve on the design of awareness activities.

Knowledge uptake was moderate across communities. Although scores were lower in communities that did not have the research team on the ground, they were still acceptable. Age did seem to affect the results in that the older youth seemed to fair better suggesting that future studies should likely adapt the activities to a more age specific target group. Although a questionnaire is not a comprehensive tool for cultural appropriateness it did allow community members to give us feedback about the acceptability of the interventions in an anonymous manner.

Tailoring interventions to local context is an important element in the success of knowledge translation strategies[13]. This project sought to capitalize on the different types of youth development resources that were available in each community. Flexibility of interventions around a common core was important for ensuring presentation of an accurate, consistent, and complete learning message while capitalizing on the unique characteristics of each community and each implementation team. Being sensitive to Inuit culture and tradition is important for ensuring that interventions are acceptable to culturally distinct communities.

This research indicates that implementation of this type of awareness activity by local implementation teams is feasible (sustained participation). This has significant resource allocation implications going forward. Achieving any awareness activities is very costly in this remote region of the country therefore knowing that it can be implemented locally is central in expanding the program in a region with few resources and therefore also speaks to its scalability.

### Limitations

Evaluating knowledge uptake posed a challenge in this project. The original plan was to use a pre-designed rubric to evaluate the content of the videos produced as part of the learning activities in order to determine knowledge uptake by the youth participants. It became apparent that many participants, particularly the younger ones, required significant coaching to help them synthesize what they learned in order to produce an educational video. This rendered the videos unusable for evaluation purposes as the content was significantly influenced by the facilitators. Furthermore our interviews were limited by only testing immediate recall after the three sessions, recall at a later date was not tested but would provide useful information. The youth that participated in the Taima TB Youth Education Initiative had widely varied abilities. Comprehension was likely influenced by education level, age, and maturity. Another challenge encountered in the evaluation of knowledge uptake was the varied ability in some participant groups to express concepts in writing. The research team made modifications to the evaluation process to overcome this issue by moving from a written evaluation to a video interview by the research team. This study would have been strengthened by a more in depth measure of the cultural appropriateness of the adapted interventions.

Next steps include evaluating the continued use and impact of these interventions. More research is needed to look at the ongoing use of these activities both in the communities where the interventions where implemented and in other communities that are looking for TB awareness building materials.

## Conclusions

The TAIMA TB youth education initiative was feasible as evidenced by the participation and attendance of youth in all communities. Feedback from local implementation teams and community members indicated that the program was acceptable for their communities. The program resulted in knowledge uptake in participants. The Territory of Nunavut is comprised of 25 communities over a vast land mass making it financially prohibitive to go to each community to deploy this initiative. However, this study has demonstrated the potential of local implementation teams to successfully deploy the Taima TB youth education initiative, which could facilitate new opportunities for sustainable TB awareness and education in other remote communities in the Arctic setting where human and financial resources are in scarce supply in a Territory like Nunavut.

### Additional Information

Additional materials for any readers interested in replicating the above study are freely available in PDF format including a facilitators manual at our website and anything that is not available at the website can be obtained by contacting our coordinator (email on website).

http://taimatb.tunngavik.com/taima-tb/taima-tb-youth-education-initiative/

## Supporting Information

S1 Appendix The 5 Things You Need To Know About TB. Messaging from the original Taima TB- Iqaluit project.
(PDF)

S2 Appendix Youth Learning Sessions. Description of youth learning sessions (Table A). Knowledge uptake results following youth learning sessions (Table B). Attendance at your learning sessions (Figure A).
(DOCX)

S3 Appendix Community Events. Description of community events (Table A). Community event attendance (Figure A).
(DOCX)
