# Supplementary material for: Developing and Field Testing a Community Based Youth Initiative to Increase Tuberculosis Awareness in Remote Arctic Inuit Communities
Source: PLoS One. 2016 Jul 14;11(7):e0159241. doi: 10.1371/journal.pone.0159241 (PMC4945095; doi:10.1371/journal.pone.0159241)
Supplement: S1 Appendix — Messaging from the original Taima TB- Iqaluit project. (PDF) [file pone.0159241.s001.pdf]

THE 5 THINGS YOU NEED TO KNOW ABOUT TB

1. TB is treated here in Nunavut and is curable.
2. People who are sick with active TB disease can have chronic cough, weight loss, night sweats or fever.
3. You may be able to infect other people if you have active TB disease in your lungs because TB spreads through the air.
4. If you are close with someone who has active TB disease, you may become infected with TB germs and develop sleeping TB infection.
5. People with sleeping TB infection are not contagious and can be treated with medication in Nunavut to prevent getting sick with active disease.

ᑕᐃᒪ ᐳᕙᓪᓗᓐᓇᖅ
TAIMA TB

www.tunngavik.com/taimatb
Taima TB Team: 867-975-4813

ᑕᓪᓕᒪᑦ

5 ᖃᐅᔨᒪᔭᕆᐊᖃᖅᑕᑎᑦ ᐳᕙᓪᓗᖕᓂᕐᒧᑦ

1 ᐳᕙᓪᓗᒃ ᑲᒪᒋᔭᐅᕙᒃᑐᖅ ᓄᓇᕗᒻᒥ ᒪᒥᓴᖅᑕᐅᔪᓐᓇᕐᓂᐊᓂᒃ.

2 ᑭᒃᑯᑐᐃᓐᓇᐃᑦ ᐋᓐᓂᐊᖅᑐᑦ ᐳᕙᓪᓗᖕᓂᕐᒧᑦ ᖃᓂᒪᒧᑦ ᐊᒃᓱᐊᓗᒃ ᖁᐃᖅᑐᖅᐸᒃᑐᑦ, ᐅᕿᒃᓚᕙᓪᓚᐊᓪᓗᑎᒃ, ᐊᐅᒪᔮᖅᐸᒃᓱᑎᒃ ᐊᐅᒃᑲᓂᖕᖓᕙᒃᓱᑎᒃ ᐅᕝᕙᓘᓐᓃᑦ ᐆᓇᖅᓱᑎᒃ.

3 ᐊᐃᑦᑐᐃᑐᐃᓐᓇᕆᐊᖃᖅᑐᑎᑦ ᐊᓯᖕᓂᑦ ᐳᕙᓪᓗᒃᑯᕕᑦ ᖃᓂᒪᒥᑦ ᐳᕙᖕᓂ ᐱᔾᔪᑎᒋᓪᓗᒍ ᐳᕙᓪᓗᖕᓂᖅ ᓯᐊᒻᒪᒃᑎᖅᐸᖕᒪᑦ ᐊᓂᕐᓂᒃᑯᑦ.

4 ᖃᓂᒋᔭᖃᕈᕕᑦ ᑭᓇᑐᐃᓐᓇᒥᑦ ᐳᕙᓪᓗᒃᐸᑦ, ᐊᐃᑦᑐᖅᑕᐅᑐᐃᓐᓇᕆᐊᖃᖅᑐᑎᑦ ᐳᕙᓪᓗᖕᓇᖅᑐᒥᑦ ᖁᐱᕐᕈᖕᒋᓐᓂᑦ ᐊᒻᒪᓗ ᑕᑯᒃᓴᐅᖕᒋᑦᑐᒥᑦ ᐳᕙᓪᓗᖕᓇᒥᑦ ᕿᖃᖅᑐᒥᑦ ᓲᕐᓗ ᐊᐃᑦᑐᖅᑕᐅᓯᒪᓗᑎᑦ.

5 ᑭᒃᑯᑐᐃᓐᓇᐃᑦ ᑕᑯᒃᓴᐅᖕᒋᑦᑐᒥᑦ ᐳᕙᓪᓗᖕᓇᒥᑦ ᕿᖃᖅᑐᒥᑦ ᓲᕐᓗ ᐊᐃᑦᑐᐃᔪᓐᓇᖕᒋᑦᑐᖅ ᐊᒻᒪᓗ ᐃᓱᐊᖅᓴᖅᑕᐅᔪᓐᓇᖅᑐᖅ ᐋᓐᓂᐊᖅᑐᖅᓯᐅᑎᓂᑦ ᐃᓱᐊᖅᓴᐅᑎᓂᑦ ᓄᓇᕗᒥ ᓄᖅᑲᖅᑎᓇᓯᐊᕐᓗᒍ ᐋᓐᓂᐊᖅᑕᐃᓕᓂᕐᒧᑦ ᑕᒪᓐᓇ ᐱᓪᓗᒍ ᐋᓐᓂᐊᕐᓇᖅ ᐆᒪᔪᖅ.

ᑕᐃᒪ ᐳᕙᓪᓗᓐᓇᖅ
TAIMA TB

www.tunngavik.com/taimatb
Taima TB Team: 867-975-4813
