# Supplementary material for: Developing and Field Testing a Community Based Youth Initiative to Increase Tuberculosis Awareness in Remote Arctic Inuit Communities
Source: PLoS One. 2016 Jul 14;11(7):e0159241. doi: 10.1371/journal.pone.0159241 (PMC4945095; doi:10.1371/journal.pone.0159241)
Supplement: S2 Appendix — Description of youth learning sessions (Table A). Knowledge uptake results following youth learning sessions (Table B). Attendance at your learning sessions (Figure A). (DOCX) [file pone.0159241.s002.docx]

**TABLE A: DESCRIPTION OF YOUTH LEARNING SESSIONS**

| Community | Primary Facilitator | Venue | Time of Day | Recruitment |
| --- | --- | --- | --- | --- |
| #1 (research team) | Youth facilitation specialist | Youth  Centre | Weekday evenings | - General invitation on radio and social media - Invitation to youth present at youth centre |
| #2 (research team) | Youth facilitation specialist | Resource portable | Weekday evenings | - General invitation on radio and social media - Invitation to youth present at youth centre |
| #3 (local implementation team) | Public Health Nurse | Classroom | During school hours | - Activities were part of cultural studies class |
| #4 (local implementation team) | Teacher | Classroom | After school | - Specific students invited to participate |

Figure A: aTTENDANCE AT YOUTH LEARNING SESSIONS

**TABLE B: KNOWLEDGE UPTAKE RESULTS FOLLOWING YOUTH LEARNING SESSIONS**

| Question | Answered correctly?  N = 14 |
| --- | --- |
| Is TB curable? (YES = 1 point, NO = 0 points) | 93% |
| Is TB treatable in <insert community>? (YES = 1 point, NO = 0 points) | 86% |
| Is sleeping TB contagious? (YES = 0 point, NO = 1 point) | 79% |
| How is TB spread or transmitted between people? Through the air (1 point) | 93% |
| What are the symptoms of active TB? (1 point per correct symptom given: chronic cough, weight loss, night sweats or fever, to maximum of 4 points) | 1 symp. = 30%  >1 symp. = 50 % |
| Can you get sleeping TB from someone who has active TB disease? (YES = 1 point, NO = 0 point) | 79% |
| Anything else you learned? (Any correct answer = 1 point) | 43% |
| TOTAL | **64%** |
