# Supplementary material for: Developing and Field Testing a Community Based Youth Initiative to Increase Tuberculosis Awareness in Remote Arctic Inuit Communities
Source: PLoS One. 2016 Jul 14;11(7):e0159241. doi: 10.1371/journal.pone.0159241 (PMC4945095; doi:10.1371/journal.pone.0159241)
Supplement: S3 Appendix — Description of community events (Table A). Community event attendance (Figure A). (DOCX) [file pone.0159241.s003.docx]

**TABLE A: DESCRIPTION OF COMMUNITY EVENTS**

| Community | Venue | Food Served | Type of Event | Activities | Event Attendance |
| --- | --- | --- | --- | --- | --- |
| #1 | Health Centre | Country food, fruit and vegetable trays | Weekday afternoon drop-in for schools classes and community members | - Presentation of TB messaging - Viewing videos - Sharing TB experiences - Q&A - Tour of TB facilities | 55 (approximately) |
| #2 | Community Hall | Country food | Weekday evening country feast | - Presentation of TB messaging - Viewing videos - Sharing TB experiences - Q&A | 106 |
| #3 | Community Hall | popcorn, some country food, fruit and vegetable trays | Saturday evening activity  Movie night theme | - Presentation of TB messaging - Viewing videos - Sharing TB experiences - Playing some of the Youth Education Initiative TB teaching games with children present | 68 |
| #4 | Community gathering place | Country food, fruit and vegetable trays | Saturday luncheon | - Presentation of TB messaging - Viewing videos - Sharing TB experiences - Q&A | 42 |

Figure A: Community Event attendance
